# Intraoperative hyponatremia is an independent predictor of one-year mortality after liver transplantation

**DOI:** 10.1038/s41598-018-37006-7

**Published:** 2018-12-21

**Authors:** Seong-Mi Yang, Sheung-Nyoung Choi, Je Hyuk Yu, Hyun-Kyu Yoon, Won Ho Kim, Chul-Woo Jung, Kyung-Suk Suh, Kook Hyun Lee

**Affiliations:** 1 Department of Anesthesiology and Pain Medicine, Seoul National University Hospital, Seoul National University College of Medicine, Seoul, Republic of Korea; 2 Department of Surgery, Seoul National University Hospital, Seoul National University College of Medicine, Seoul, Republic of Korea

## Abstract

Preoperative hyponatremia is associated with an increased risk of mortality on the liver transplantation (LT) waiting list. We sought to investigate the impact of pre- and intraoperative serum sodium levels on the one-year mortality after LT. We identified 1,164 patients for whom preoperative and intraoperative serum sodium levels were available. Cox regression analysis with multivariable adjustment was performed for one-year mortality. A propensity score matching analysis was performed for preoperative and intraoperative serum sodium groups to compare one-year survival. The cutoff of sodium level with minimal p-value was 130 mEq/L for both preoperative and intraoperative sodium. Intraoperative hyponatremia was an independent predictor of one-year mortality in the multivariable Cox regression analysis, while preoperative hyponatremia was not. Kaplan-Meier curve showed that there was a significant difference in the one-year mortality between preoperative and intraoperative serum sodium groups. However, after propensity score matching, there was no difference in the one-year mortality among the preoperative sodium groups, while there was a significant difference among the intraoperative sodium groups. Intraoperative hyponatremia defined by mean sodium <130 mEq/L was independently associated with a significantly high one-year mortality. Mean intraoperative serum sodium levels may be a better prognostic predictor than preoperative serum sodium levels.

## Introduction

Hyponatremia is a common yet ominous sign in patients with end-stage liver disease (ESLD)^1,2^. Almost half of the patients with cirrhosis have serum sodium concentrations below the lower limit of the normal range (<135 mEq/L)^3^. Hyponatremia in patients with ESLD is associated with increased mortality independently of other indicators of liver disease severity such as the Model for End-Stage Liver Disease (MELD) score^2,4–10^. Also, preoperative hyponatremia of <130 mEq/L was associated with decreased post-liver transplantation (LT) survival^11,12^. Meanwhile, several studies also reported no significant association between preoperative hyponatremia and postoperative mortality^13–15^. Furthermore, there was no difference in the 6-month survival rate between those for whom MELD-Na score was used for liver allocation and those for whom the standard MELD score was used^16^. Therefore, the association of preoperative hyponatremia with postoperative mortality continue to be debated.

Additionally, the impact of intraoperative hyponatremia on post-LT mortality has not been evaluated. A previous retrospective study reported that a larger increase in serum sodium concentration after surgery compared to preoperative baseline concentrations was associated with worse recipient short-term outcomes^17^. However, in this study, the impact of intraoperative hyponatremia during transplantation surgery was not evaluated. Intraoperative hyponatremia may be more closely related to the posttransplant outcomes than preoperative hyponatremia because the intraoperative serum sodium fluctuates more due to administration of a large amount of fluid, sodium bicarbonate, and diuretics. During surgery, frequent preload change occurs by clamping of inferior vena cava and bleeding and metabolic acidosis develops during anhepatic and reperfusion of liver graft. Therefore, these significant change in sodium homeostasis during surgery may be more strongly associated with postoperative mortality compared to preoperative serum sodium levels.

Additionally, among the studies reporting the impact of hyponatremia on mortality in patients with ESLD, the level of serum sodium used to define hyponatremia does not seem to be standardized. Hyponatremia in patients with decompensated cirrhosis was defined as a serum sodium level <130 mEq/L in previous studies^2,18^, although the lower limit of normal serum sodium is 135 mEq/L. Accordingly, many studies use <130 mEq/L to define hyponatremia^11,12,14^, while other studies still use <135 mEq/L as a cutoff of hyponatremia^13,19^. However, there have been no studies that attempted to find optimal cutoff of serum sodium that is associated with increased risk of post-LT mortality.

Therefore, in this study, we attempted to conduct a retrospective observational study (1) to address whether preoperative and intraoperative hyponatremia during LT are associated with diminished post-LT survival and (2) to find the optimal cutoff of preoperative and intraoperative serum sodium concentration that is associated with poor posttransplant survival.

## Results

### Recipient characteristics

A flow diagram showing the inclusion of participants in the study and the results of propensity score matching are shown in Supplemental Fig. S1. There were 1180 primary transplant recipients who met the inclusion criteria. Patients who were hypernatremic (mean pre- or intraoperative sodium ≥145 mEq/L) were excluded from the final analysis (n = 16) due to the low incidence. Patient characteristics are compared between the patients with mean intraoperative sodium level <130 mEq/L and those with mean intraoperative sodium levels of 130–145 mEq/L in Table 1. This cutoff was determined according to the minimal p-value approach described later. The distribution of sodium levels is shown in Supplemental Fig. S2. Preoperative sodium levels were less than 130 mEq/L in 221 patients (19.0%) and mean intraoperative sodium values were less than 130 mEq/L in 338 patients (29.0%). The median (interquartile range) of preoperative sodium was 137 mEq/L (132–141), and those of mean intraoperative sodium was 134 mEq/L (129–137). There were significant differences in the baseline medical conditions and intraoperative variables between the intraoperative sodium groups. Patients with intraoperative hyponatremia (<130 mEq/L, n = 338, 29.0%) had frequent diabetes mellitus, frequent alcoholic liver cirrhosis, lower preoperative hemoglobin level, lower albumin level, higher MELD score, higher Child classification, longer cold ischemic time, frequent red blood cell and fresh frozen plasma transfusion than those with mean serum sodium between 130 and 145 mEq/L.

**Table 1:** Patient characteristics and perioperative parameters according to intraoperative mean sodium groups.

Characteristic	Hyponatremia (<130 mEq/L)	Normonatremia (130–145 mEq/L)	P-value
Patient population, hyperglycemia corrected	338 (29.0%)	826 (71.0%)	
Donor type			
Deceased donor, n	133 (39.3)	234 (28.3)	<0.001
Living donor, n	205 (60.7)	592 (71.7)	<0.001
Demographic data			
Age, years	54 (48–60)	53 (47–60)	0.310
Female, n	101 (31.1)	224 (27.1)	0.340
Body-mass index, kg/m^2^	22.8 (20.7–25.4)	23.2 (21.3–25.3)	0.097
Background medical status			
Hypertension, n	34 (10.1)	89 (10.8)	0.718
Diabetes mellitus, n	66 (19.5)	107 (13.0)	0.004
Alcoholic liver cirrhosis, n	59 (17.5)	71 (8.6)	<0.001
HBV hepatitis, n	151 (44.7)	326 (39.5)	0.101
HCV hepatitis, n	32 (9.5)	66 (8.0)	0.410
Hepatocellular carcinoma, n	106 (31.4)	363 (43.9)	<0.001
Cholestatic disease, n	10 (3.0)	18 (2.2)	0.426
Preoperative hemoglobin, g/dl	10.0 (9.0–11.7)	11.0 (9.3–12.8)	<0.001
Preoperative serum albumin level, mg/dl	2.8 (2.4–3.2)	3.0 (2.6–3.5)	<0.001
Hepatorenal syndrome, n	49 (14.5)	10 (1.2)	<0.001
MELD score	17.0 (12.6–24.0)	14.8 (10.4–20.3)	<0.001
CTP score	8 (7–11)	8 (6–10)	<0.001
Child class, n (A/ B/ C)	31 (15.1)/ 94 (45.9)/ 80 (39.0)	187 (31.6)/ 258 (43.6)/ 147 (24.8)	<0.001
Preoperative LVEF. %	65 (62–68)	65 (62–68)	0.608
Preoperative beta-blocker, n	13 (3.8)	46 (5.6)	0.133
Preoperative diuretics, n	17 (5.0)	28 (3.4)	0.189
Donor/ graft factors			
Age, years	30 (23–39)	30 (24–39)	0.864
Estimated GRWR	1.20 (1.06–1.42)	1.20 (1.04–1.41)	0.360
ABO incompatible, n	2 (0.6)	30 (3.6)	0.004
Operation and anesthesia details			
Operation time, hour	6.5 (5.5–7.6)	6.7 (5.6–7.7)	0.543
Cold ischemic time, min	92 (71–240)	82 (67–230)	<0.001
Warm ischemic time, min	30 (29–35)	30 (26–35)	0.230
Intraoperative mean blood glucose, mg/dl	164 (147–180)	162 (143–180)	0.339
Crystalloid administration, ml/kg	3950 (2800–5100)	3490 (2300–5300)	0.036
Colloid administration, ml/kg	0 (0–500)	0 (0–500)	0.154
Intraoperative furosemide, mg	0 (0–10)	0 (0–10)	0.326
Intraoperative sodium bicarbonate, mEq	90 (0–180)	100 (0–222)	0.120
Bleeding and transfusion amount			
pRBC transfusion, units	8 (4–12)	6 (2–12)	<0.001
FFP transfusion, units	8 (3–12)	5 (0–12)	0.002
Blood loss per body weight, ml/kg	52 (30–91)	43 (22–102)	0.021

The values are expressed as the median [interquartile range] or number (%).

CTP score = Child-Turcotte-Pugh score, LVEF = left ventricular ejection fraction, GRWR = graft versus recipient body weight ratio, p-RBC = packed red blood cells, FFP = fresh frozen plasma.

### Association between intraoperative sodium and mortality

To evaluate the cutoff of pre- and intraoperative sodium levels that is associated with one-year mortality, the minimal p-value approach was used. The results of serial multivariable Cox regression analyses according to the different cutoffs of pre- and intraoperative serum sodium are shown in Table 2 (for one-year mortality). Mean intraoperative sodium <130 mEq/L showed the least p-value (p = 0.006) and the largest hazard ratio (HR 2.34, 95% confidence interval [CI], 1.32–4.11). Preoperative serum sodium <130 was significantly associated with one-year mortality (HR 1.36, 95% CI 1.02–1.87, p = 0.044).

**Table 2:** Adjusted hazard ratios (95% confidence intervals) and its P-values according to the categorized preoperative and intraoperative mean sodium levels with different cutoffs determined by multivariable Cox regression analysis for one-year mortality.

Cutoff	Preoperative sodium			Intraoperative sodium		
	HR	95% CI	P-value	HR	95% CI	P-value
Continuous	1.00	0.91–1.03	0.302	0.95	0.93–0.97	<0.001
<140	0.84	0.53–1.34	0.488	0.63	0.23–1.70	0.440
<135	1.11	0.75–1.63	0.712	1.92	0.94–3.90	0.082
<130	1.36	1.02–1.87	0.044	2.34	1.32–4.11	0.006
<125	0.79	0.45–1.33	0.383	1.98	1.07–3.65	0.030
<120	0.61	0.20–1.89	0.457	0.30	0.18–1.95	0.189

HR = hazard ratio, CI = confidence interval.

The following variables were used for adjustment: living versus deceased donor, recipient age, sex, body-mass index, history of hypertension, diabetes mellitus, preoperative diuretics administration, hepatorenal syndrome, cold-ischemic time, warm ischemic time, MELD score, graft-recipient body weight ratio (GRWR), ABO-incompatibility transplantation, preoperative left ventricular ejection fraction, operation time, red blood cell transfusion during surgery, intraoperative mean blood glucose, preoperative hemoglobin, and preoperative albumin.

Using the cutoff of 130 mEq/L of pre- and intraoperative hyponatremia, both preoperative and intraoperative hyponatremia was entered into the multivariable Cox regression analysis for one-year mortality and logistic regression analysis for in-hospital mortality. The result of Cox regression analysis is shown in Table 3. Harrell’s c was 0.75 and Somer’s D was 0.55. Preoperative serum sodium <130 mEq/L was not independently associated with one-year all-cause mortality, while intraoperative mean sodium <130 mEq/L was independently associated with one-year mortality (adjusted Hazard ratio 2.34, 95% confidence interval [CI] 1.32–4.11). The results of logistic regression analysis are shown in Supplemental Table S1. Intraoperative serum sodium <130 mEq/L was independently associated with in-hospital mortality, while preoperative hyponatremia was not.

**Table 3:** Cox proportional hazard regression analysis for one-year mortality.

Variable	Adjusted Hazard Ratio	95% CI	P-value
Age, recipient, per 10 year	1.33	1.14–1.62	0.001
MELD score	1.14	1.02–1.06	0.003
Preoperative albumin, g/dL	1.03	0.79–1.22	0.060
Preoperative hemoglobin, g/dL	0.88	0.80–0.95	0.010
Operation time, per 1 hour	1.16	1.06–1.27	0.001
Intraoperative mean sodium <130 mEq/L	2.34	1.32–4.11	<0.001

MELD score = model for end-stage liver disease score, LVEF = left ventricular ejection fraction, CI = confidence interval.

Stepwise backward variable selection process was used with a cutoff of *P* < 0.10.

The following variables were used for adjustment: living versus deceased donor, recipient age, sex, body-mass index, history of hypertension, diabetes mellitus, preoperative diuretics administration, hepatorenal syndrome, cold-ischemic time, warm ischemic time, MELD score, graft-recipient body weight ratio (GRWR), ABO-incompatibility transplantation, preoperative left ventricular ejection fraction, operation time, red blood cell transfusion during surgery, intraoperative mean blood glucose, preoperative hemoglobin, and preoperative albumin.

### Mean intraoperative serum sodium and Post-LT Survival

**Figure 1:**
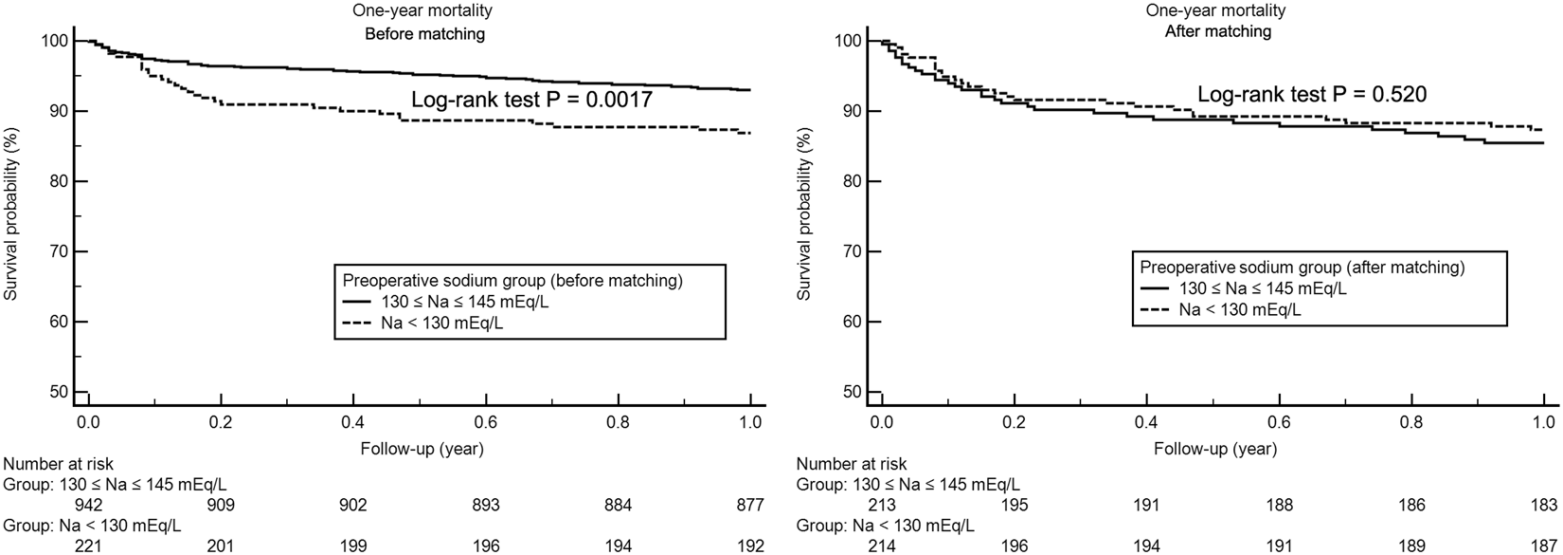
Kaplan-Meier survival curve analysis according to preoperative serum sodium groups (Na < 130 mEq/L and 130 ≤ Na ≤ 145 mEq/L) before and after propensity score matching.

**Figure 2:**
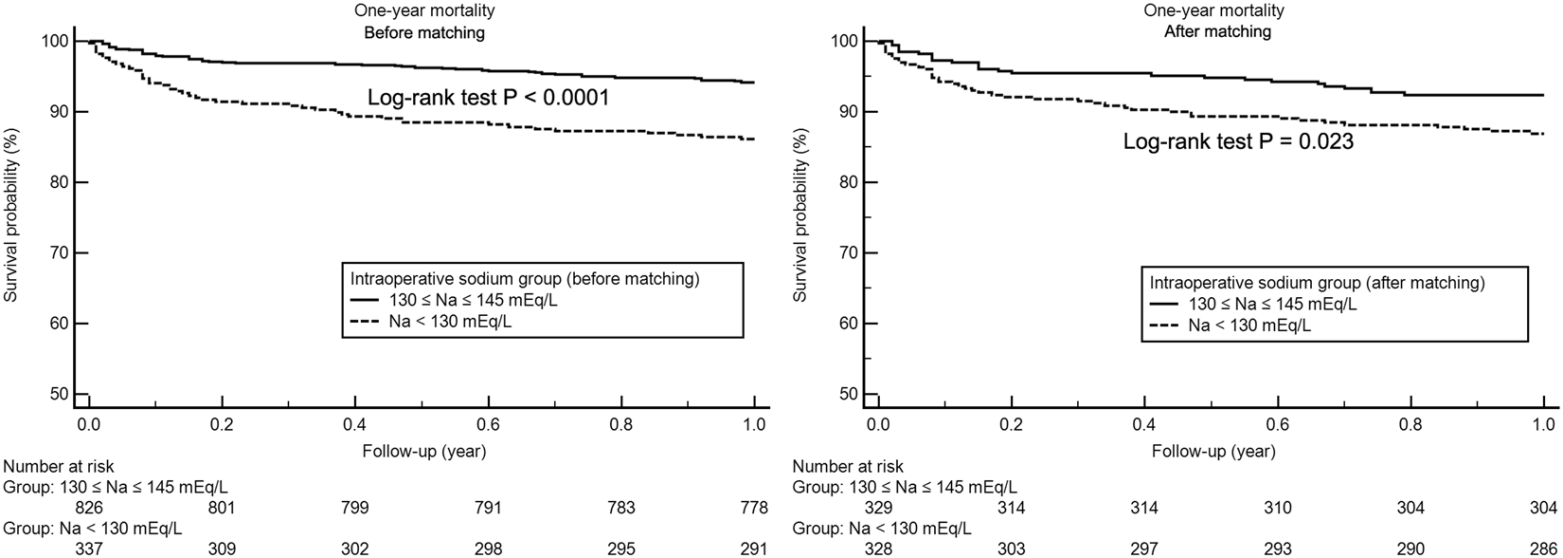
Kaplan-Meier survival curve analysis according to intraoperative serum sodium groups (Na < 130 mEq/L and 130 ≤ Na ≤ 145 mEq/L) before and after propensity score matching.

The median follow-up after LT was 365 days (interquartile range, 365-365 days). 112 (9.6%) of the 1164 patients (9.6%) died in-hospital, and 132 patients (11.3%) died within one year after LT. Figures 1 and 2 illustrates patient survival up to one year after LT before and after propensity score matching. It is apparent that there is a significant difference in the survival between both preoperative and intraoperative hyponatremic (<130 mEq/L) and normonatremic (130–145 mEq/L) patients, suggesting the survival of hyponatremic patients to be significantly shorter (preoperative: Log-rank test, p = 0.0017, chi-square = 9.8; intraoperative: Log-rank test, p < 0.0001, chi-square = 26.5). However, after propensity score matching, there was a significant difference in survival between only the intraoperative sodium groups (Log-rank test, p = 0.023, chi-square = 9.28), while not between the preoperative sodium groups (Log-rank test, p = 0.520, chi-square = 0.41).

Table 4 shows the comparison of the secondary clinical outcomes after liver transplantation according to the intraoperative mean sodium groups. There were significant differences in the incidence of postoperative hemodialysis, length of intensive care unit and hospital stay. Table 5 shows the comparison of the cause of death during one year after transplantation according to the intraoperative mean sodium groups. The incidences of hepatic failure, recurred hepatocellular carcinoma and renal failure were significantly higher in the hyponatremia group than the normonatremia group.

**Table 4:** Secondary clinical outcomes after liver transplantation according to intraoperative mean serum sodium levels before and after propensity score matching.

	Before propensity score matching			After propensity score matching		
	Hyponatremia (<130 mEq/L) (n = 338)	Normonatremia (130–145 mEq/L) (n = 826)	P-value	Hyponatremia (<130 mEq/L) (n = 329)	Normonatremia (130–145 mEq/L) (n = 329)	P-value
Postoperative hemodialysis, n	57 (16.9)	72 (8.7)	<0.001	56 (17.0)	36 (10.9)	0.025
Osmotic demyelination syndrome, n	7 (2.1)	—	0.090	6 (1.8)	—	0.083
In-hospital mortality, n	55 (16.3)	57 (6.9)	<0.001	51 (15.5)	28 (8.5)	0.006
One-year mortality, n	63 (18.6)	69 (8.4)	<0.001	59 (17.9)	32 (9.7)	0.002
Length of ICU stay, days	5 (4–8)	5 (4–7)	0.002	5 (4–7)	5 (4–8)	0.012
Length of hospital stay, days	20 (15–28)	17 (14–28)	0.049	21 (16–30)	17 (14–28)	<0.001

Data are presented as the number (%) or median [interquartile range] or number (%). ICU = intensive care unit.

**Table 5:** Comparison of cause of death during one year after transplantation according to the intraoperative serum sodium groups before and after matching.

Cause of death	Before propensity score matching			After propensity score matching		
	Hyponatremia (<130 mEq/L) (n = 338)	Normonatremia (130–145 mEq/L) (n = 826)	P-value	Hyponatremia (<130 mEq/L) (n = 329)	Normonatremia (130–145 mEq/L) (n = 329)	P-value
Hepatic failure, n	20 (4.8)	5 (0.7)	<0.001	14 (4.3)	5 (1.5)	0.036
Hapatitis, n	8 (1.9)	4 (0.5)	0.027	6 (1.8)	3 (0.9)	0.314
Liver abscess, n	4 (1.0)	1 (0.1)	0.060	2 (0.6)	1 (0.3)	0.563
Recurred hepatocellular carcinoma, n	37 (8.8)	28 (3.8)	<0.001	30 (9.1)	30 (4.6)	0.021
Malignancy other than liver, n	3 (0.7)	2 (0.3)	0.264	2 (0.6)	1 (0.3)	0.563
Renal failure, n	8 (1.9)	1 (0.1)	0.032	6 (1.8)	1 (0.3)	0.042
Cerebrovacular accident, n	—	2 (0.3)	0.168	—	1 (0.3)	0.999
Pulmonary thromboembolism, n	1 (0.2)	—	0.999	1 (0.3)	—	0.999
Miscellaneous, n	3 (0.7)	5 (0.7)	0.933	3 (0.9)	4 (1.2)	0.704

Data are presented as number (%).

Supplemental Figure S3 shows the results of the Kaplan-Meier survival curve analysis according to first intraoperative serum sodium groups (Na < 130 mEq/L and 130 ≤ Na ≤ 145 mEq/L) before and after propensity score matching. There were significant differences between the sodium groups before and after matching.

## Discussion

We investigated the association between preoperative and intraoperative serum sodium levels and one-year all-cause mortality after liver transplantation. The main finding of our analysis was that intraoperative serum hyponatremia defined as <130 mEq/L was an independent predictor of one-year mortality after adjustment of MELD score and other perioperative covariates of the severity of illness. This cutoff was determined according to the minimal p-value approach. Although preoperative hyponatremia was also associated with poor one-year survival, the association was not significant after propensity score matching and was not an independent predictor of one-year mortality in the multivariable Cox regression analysis. Therefore, our data indicated that intraoperative hyponatremia determined by a mean value < 130 mEq/L may be a stronger prognostic factor that is associated with one-year mortality after liver transplantation.

It has gained consensus that preoperative hyponatremia adversely affects the survival of end-stage liver patients, including those on the LT wait list. However, the impact of preoperative hyponatremia on post-LT survival has been debated. While several studies reported a significant association between preoperative hyponatremia and decreased short-term post-LT survival^11,12^, other studies reported no impact of preoperative hyponatremia on survival^13,14^. A recent large retrospective study of 47,254 patients who underwent LT in the United States reported no association of preoperative hyponatremia and 90-day mortality after LT^15^.

We hypothesized that the mean intraoperative serum sodium levels may be significantly and independently associated with one-year posttransplant mortality. As shown in our results, the intraoperative serum sodium levels tended to be lower than the preoperative value. Despite frequent metabolic acidosis during surgery and sodium bicarbonate administration, a large amount of crystalloid and colloid administration by hemodynamic derangement during surgery may lead to dilutional hyponatremia. Also, the amount of fluid administration tend to be excessive before declamping of inferior vena cava, and diuretics used to treat fluid overload may also contribute to intraoperative hyponatremia. This frequent and severe hyponatremia during surgery may be strongly associated with postoperative mortality than preoperative hyponatremia.

There could be another explanation for the strong association between intraoperative hyponatremia and postoperative mortality. First, the preoperative serum sodium levels investigated in previous studies were obtained at a single time point with varied interval from the time of transplantation^9,13,15,19^. Preoperative sodium levels obtained in our study was also from a single measurement in most of the cases. Meanwhile, intraoperative serum sodium levels were obtained during standardized pre-specified time points during surgery in our study. Mean sodium levels calculated from eight intraoperative values contain information of sodium homeostasis during the surgery including the important periods of graft anastomosis and reperfusion. Mean sodium levels may reflect the overall status of sodium homeostasis during LT with high validity. Second, although preoperative serum sodium concentration may vary depending on the factors including intravascular volume status and the amount of administered diuretics at the time of measurements, previous studies did not extensively adjust the potential covariates through propensity score matching^14–17^. Adjustment of many confounding factors may have revealed the more strong association between intraoperative sodium levels and mortality.

It is not clear whether the association between serum sodium and mortality is attributable to hyponatremia per se or if it simply reflects the fact that patients with poorly maintained serum sodium concentration during the transplantation surgery tend to have a baseline and operation-related characteristics that are related to poor patient survival. Hyponatremia is associated with hepatorenal syndrome, ascites, and mortality from liver disease^20–22^. Our analysis of the cause of death during one year after transplantation showed that the incidence of hepatic failure and renal failure were significantly higher in the patients with intraoperative hyponatremia, which was the same in the propensity score-matched cohort. This means that intraoperative hyponatremia may be associated with the prognosis of liver graft and risk of postoperative renal failure. Furthermore, our sensitivity analysis of first intraoperative sodium groups showed that there was a significant difference in survival between groups. This difference was also observed after propensity score matching. This result supports the hypothesis that the intraoperative sodium levels may be more important to predict one-year mortality than the preoperative serum sodium level.

Specifically, intraoperative hyponatremia is associated with postoperative central pontine myelinolysis that could influence the overall mortality^23^. More patients in the intraoperative hyponatremia group developed osmotic demyelination syndrome than normonatremia group. The comparison of baseline characteristics of our study population according to mean intraoperative sodium levels showed significant differences in the baseline medical status of patients. The patients with mean hyponatremia had higher MELD scores, received large crystalloid infusions and had more frequent intraoperative transfusions. Although these covariates were adjusted to evaluate the association and were mated using propensity score, our results may have been biased by other unknown or unmeasured baseline and intraoperative characteristics.

To our knowledge, there is no previous study that reported the prognostic value of mean intraoperative serum sodium. A previous small retrospective study of 164 patients evaluated the influence of a change in serum sodium after surgery on the clinical outcomes after LT^17^. They compared 30-day and one-year mortality according to the delta sodium, a difference between pre- and postoperative serum sodium levels. A larger increase in serum sodium was associated with prolonged intubation time, and ICU stay, but not mortality.

The present study has several important limitations. First, this was a single-center retrospective study of a relatively small sample size. Our data showed that intraoperative serum sodium levels tend to become lower than the preoperative value during transplantation surgery. This may be because the patients become sicker at the time of transplant and/or fluid administration during anesthesia induction may cause hemodilution and a decrease in serum sodium concentration. With the same sample size, the more frequent intraoperative hyponatremia may be strongly associated with mortality compared to preoperative hyponatremia. Second, our data came from the institutional database of more than ten years. The policy of administrating colloid, sodium bicarbonate or diuretics, and the choice of the type of main crystalloid solution may have changed over this extended period. Third, the clinical implication of our finding of an association between intraoperative hyponatremia and mortality is uncertain. A recent study reported that patients with hyponatremia corrected before LT had no survival benefit compared with the patients with normal sodium homeostasis^14^. However, little is known about how the correction of intraoperative hyponatremia affects long-term outcomes after LT, except the risk of rapid correction of hyponatremia.

In conclusion, the present study demonstrated that intraoperative hyponatremia independently predicts one-year mortality after LT, while preoperative hyponatremia did not. The cutoff of hyponatremia during LT was determined to be 130 mEq/L according to the minimal p-value approach. Intraoperative mean serum sodium levels may have prognostic value independent of other predictors of patient survival.

## Methods

### Data source

This retrospective observational study was approved by the institutional review board of Seoul National University Hospital (1803-096-930). All methods were carried out in accordance with the approved guidelines. The need for informed consent was waived given the study’s retrospective design. We retrospectively reviewed the prospectively collected liver transplantation data base of our institution of 1211 consecutive adult patients (≥18 years old) who underwent liver transplantation for the first time at our institution between January 2005 and December 2015. Patients who did not have preoperative or intraoperative serum sodium concentrations reported (n = 31) were excluded. The remaining 1180 patients were included.

### Anesthesia, Surgical technique and immunosuppression

During the study period, organs for non-living related transplant were allocated according to the policy of Korea Network for Organ Sharing (KONOS). According to the rules of KONOS, organs are allocated according to the grading of emergency. The grade of emergency ranges from 1 to 5. The emergency grade 1 is determined when fulminant hepatic failure develops in a patient admitted in the intensive care unit with more than one of the following conditions including ventilator therapy, hemodialysis and prolonged prothrombin time: international normalized ratio (>2.0). The grade 1 is determined regardless of the MELD score. The emergency grades 2 to 5 is determined according to the MELD score: grade 2 (MELD 38 to 40), grade 3 (MELD 31 to 37), grade 4 (MELD 21 to 30), grade 5 (MELD < 20).

The anesthesia protocol of our institution during the study period was as follows. Anesthesia was induced with propofol and rocuronium, and was maintained with sevoflurane and remifentanil. Volume controlled ventilation was maintained with a tidal volume of 6–8 ml/kg and a FiO_2_ of 0.5. Radial and femoral arterial pressures were continuously monitored. A Swan-Ganz catheter was inserted through a 9 Fr Advanced Venous Access catheter (Edward Lifesciences, Irvine, CA) that was placed into the right internal jugular vein. Continuous cardiac index and right ventricle-associated variables were monitored using the Vigilance II monitor (Edward Lifesciences, Irvine, CA). Ephedrine and continuous infusion of dopamine and/or norepinephrine and/or epinephrine were used to maintain hemodynamics according to the monitored cardiac index, mixed venous oxygen saturation, and systemic vascular resistance. Donor grafts were prepared with histidine–tryptophan–ketoglutarate solution. The piggyback technique was used to anastomose the graft and donor vessels. End-to-end anastomosis of the hepatic artery and duct-to-duct anastomosis of the bile duct were carried out in succession. During the surgery, immunosuppression was induced with 1000 mg methylprednisolone (Solumedrol, Pfizer, Ballerup, Denmark) and 20 mg basiliximab I.V. (Simulect, Novartis Pharma B.V., Arnhem, Netherlands). Intraoperative diuretics of furosemide 10 to 20 mg were used in patients with persistent positive fluid balance and when volume overload was suspected with pulmonary artery occlusion pressure being greater than 18 mmHg. Sodium bicarbonate of 40 ml was administered when base excess was less than −10 mEq/L. Vasopressin was not used intraoperatively during the study period.

### Data collection

Demographic, clinical, and laboratory data at the time of LT were extracted from our liver transplantation database including MELD score and underlying liver disease diagnoses. Preoperative serum sodium levels measured within one month before transplantation were collected. If there were more than one measurements of serum sodium during the month before surgery, the average value was used for preoperative serum sodium. Mean intraoperative serum sodium levels were calculated as an average of the following eight values routinely measured according to our anesthesia protocol of our institution: after anesthesia induction, 1 hour after anesthesia induction, 10 min after the beginning of the anhepatic phase, 5 min before and after graft reperfusion, 20 min after reperfusion, 5 min after the start of biliary reconstruction, and at the end of surgery. All perioperative and intraoperative serum sodium levels were corrected for hyperglycemia^24^. Hyperglycemia-corrected serum sodium levels were used to classify and match the different sodium groups.

The primary outcome variable was one-year all-cause mortality after liver transplantation. Secondary outcomes included in-hospital mortality, the incidence of osmotic demyelination syndrome, length of ICU and hospital stay, and the incidence of postoperative renal replacement therapy. Patient survival was calculated as the interval between LT and the last known follow-up or death.

### Data analysis

SPSS software version 23.0 (IBM Corp., Armonk, NY, USA) and STATA/MP version 15.1 (StataCorp, College Station, TX, USA) were used to analyze the data. For all analyses, *P* < 0.05 was considered statistically significant. The Shapiro-Wilk test was used to determine the normality of the data.

First, we determined the appropriate serum sodium cutoff level that is associated with posttransplant all-cause 1-yr mortality. A minimal p-value approach was used to determine the cutoff of sodium levels with a range of 120 to 140 mEq/L. We did not evaluate cutoff >140 because we intended to find the cutoff of hyponatremia. Multivariable Cox proportional hazard regression analyses were performed using different cutoffs for both preoperative and intraoperative serum sodium, separately. Cox regression analyses included the following covariates that may be associated with postoperative mortality: living versus deceased donor, recipient age, sex, body-mass index, history of hypertension, diabetes mellitus, preoperative diuretics administration, hepatorenal syndrome, cold-ischemic time, warm ischemic time, MELD score, graft-recipient body weight ratio, ABO-incompatibility transplantation, preoperative left ventricular ejection fraction, operation time, red blood cell transfusion during surgery, intraoperative mean blood glucose, preoperative hemoglobin and preoperative albumin.

Second, we attempted to evaluate the association between preoperative and mean intraoperative sodium levels and postoperative in-hospital and one-year mortality. Multivariable logistic regression analysis was performed for in-hospital mortality, while multivariable Cox regression analysis was performed for one-year mortality. Both analyses included the same covariates listed above and also included preoperative and intraoperative hyponatremia using the cutoff determined by the above analysis.

Third, a propensity score matching analysis was performed between hyponatremia and normonatremia groups for both preoperative and intraoperative serum sodium levels using the cutoffs determined by the minimal p-value approach. This analysis was performed to reduce any confounding factors that contribute to the postoperative morality. After propensity score matching, the survival between the different sodium groups and the secondary outcomes variables were compared between the matched groups using the Kaplan-Meier curve. Multivariable logistic regression analysis was used to determine the probabilities of hyponatremia and normonatremia group assignment, which was used for matching. The following covariates were considered for the propensity score matching: living versus deceased donor transplantation, recipient age, sex, body-mass index, history of hypertension, diabetes mellitus, preoperative diuretics administration, hepatorenal syndrome, cold-ischemic time, warm ischemic time, MELD score, graft-recipient body weight ratio, ABO-incompatibility transplantation, preoperative left ventricular ejection fraction, operation time, red blood cell transfusion during surgery, intraoperative mean blood glucose, preoperative hemoglobin and preoperative albumin.

Patients were matched 1:1 using a greedy nearest neighbor algorithm with a caliper of 0.1 standard deviations of the logit-transformed propensity score. A total of 428 patients were matched in the preoperative serum sodium groups while 658 patients were matched between the two intraoperative serum sodium groups (Supplemental Figs S4 and S5). The Nagelkerke’s R^2^ of regression model were 0.31 and 0.30 for preoperative and intraoperative matching, respectively. There was no imbalance between the matched groups (standardized difference >0.1 represents imbalance).

Fourth, a Kaplan-Meier curve analysis of one-year mortality was performed for both the preoperative and intraoperative serum sodium groups. The log-rank test was used to determine statistical significance between the different serum sodium groups.

Fifth, as a sensitivity analysis, we evaluated the association between first intraoperative sodium levels and one-year mortality. We performed a propensity score matching between first intraoperative sodium groups. A Kaplan-Meier curve analysis of one-year mortality was performed between the sodium groups before and after matching.

The missingness for age, sex, body-mass index, MELD components, serum sodium, length of hospital stay, and mortality was 0%. Missing values of other covariates were less than 8%. We imputed the missing values according to the incidence of missing. If the incidence of missing was <3%, the missing was substituted by the mean for continuous variable and by the mode for incidence variable. Missing values of variables with a ratio of missing was >3% and <8% were replaced by hot-deck imputation.

## Supplementary information

Supplementary materials.
