# Supplementary material for: Intraoperative hyponatremia is an independent predictor of one-year mortality after liver transplantation
Source: Sci Rep. 2018 Dec 21;8:18023. doi: 10.1038/s41598-018-37006-7 (PMC6303312; doi:10.1038/s41598-018-37006-7)

**Intraoperative hyponatremia is an independent predictor of one-year mortality after liver transplantation**

**: Supplemental materials**

**Seong-Mi Yang**, MD^1*^, **Sheung-Nyoung Choi**, MD^1*^, **Je Hyuk Yu**, MD^1^, **Hyun-Kyu Yoon**, MD^1^, **Won Ho Kim**, MD,PhD^1^, **Chul-Woo Jung**, MD,PhD^1^, **Kyung-Suk Suh**, MD,PhD^2^, **Kook Hyun Lee**, MD,PhD^1^

^1^Department of Anesthesiology and Pain Medicine, Seoul National University Hospital, Seoul National University College of Medicine, Seoul, Republic of Korea

^2^Department of Surgery, Seoul National University Hospital, Seoul National University College of Medicine, Seoul, Republic of Korea

*These two authors contributed equally as co-first authors.

| List | Title | Page |
| --- | --- | --- |
| **Supplemental Table S1** | Multivariable logistic regression analysis for in-hospital mortality. | **3** |
| **Supplemental Figure S1** | Flow diagram showing inclusion and exclusion of study participants for the analysis of preoperative (upper) and intraoperative (lower) serum sodium levels. | **4** |
| **Supplemental Figure S2** | Distribution of preoperative and mean intraoperative sodium levels. | **6** |
| **Supplemental Figure S3** | Line plot and and histograms showing standardized mean differences before and after propensity score matching of two preoperative serum sodium groups | **7** |
| **Supplemental Figure S4** | Line plot and histograms showing standardized mean differences before and after propensity score matching of two intraoperative mean serum sodium groups | **8** |
| **Supplemental Figure S5** | Kaplan-Meier survival curve analysis according to first intraoperative serum sodium groups (Na <130 mEq/L and 130 ≤ Na ≤ 145 mEq/L) before (upper) and after propensity score matching (lower). | **9** |

**Supplemental Table S1.** Multivariable logistic regression analysis for in-hospital mortality.

| Variable | Adjusted Odds Ratio | 95% CI | p-value |
| --- | --- | --- | --- |
| Age, recipient, per 10 year | 1.32 | 1.08 – 1.68 | 0.008 |
| MELD score | 1.04 | 1.03 – 1.07 | <0.001 |
| Preoperative albumin, g/dL | 1.21 | 0.94 – 1.57 | 0.125 |
| Preoperative hemoglobin, g/dL | 0.93 | 0.82 – 1.04 | 0.175 |
| Operation time, per 1 hour | 1.16 | 1.04 – 1.31 | 0.006 |
| Intraoperative mean sodium <130 mEq/L | 3.25 | 2.12 – 5.12 | <0.001 |

MELD score = model for end-stage liver disease score, CI = confidence interval.

Stepwise backward variable selection process was used with a cutoff of *P* < 0.10.

The following variables were considered for multivariable analysis: recipient age, sex, body-mass index, history of hypertension, diabetes mellitus, preoperative diuretics administration, hepatorenal syndrome, cold-ischemic time, warm ischemic time, MELD score, graft-recipient body weight ratio, ABO-incompatibility transplantation, preoperative left ventricular ejection fraction, operation time, red blood cell transfusion during surgery, preoperative hemoglobin, and preoperative albumin.

**Supplemental Figure S1.** Flow diagram showing inclusion and exclusion of study participants for the analysis of preoperative (upper) and intraoperative (lower) serum sodium levels. Hyperglycemia-corrected sodium levels were used to classify and match the different sodium groups.

All patients who underwent

liver transplantation

during between 2004 and 2015 (n= 1211)

Excluded:

Missing in sodium data (n = 31)

Remaining (n =1180)

Preoperative Hypernatremia (Na > 145)

(n = 15): excluded from the final analysis

Preoperative

Normonatremia (130 ≤ Na ≤145)

(n = 943)

Preoperative

Hyponatremia (< 130 mEq/L)

(n = 221)

Preoperative

Normonatremia (130 ≤ Na ≤145)

(n = 214)

Preoperative

Hyponatremia (< 130 mEq/L)

(n = 214)

Propensity score matching (1:1)

: demographics, baseline medical condition, surgery-related parameters

Intraoperative

Hyponatremia (< 130 mEq/L)

(n = 338)

Intraoperative Hypernatremia (Na > 145)

(n = 16): excluded from the final analysis

Remaining (n =1180)

Intraoperative

Normonatremia (130 ≤ Na ≤145)

(n = 826)

Propensity score matching (1:1)

: demographics, baseline medical condition, surgery-related parameters

Intraoperative

Hyponatremia (< 130 mEq/L)

(n = 329)

Intraoperative

Normonatremia (130 ≤ Na ≤145)

(n = 329)

**Supplemental Figure S2**. Distribution of preoperative and mean intraoperative sodium levels. Hyperglycemia-corrected sodium levels were used.


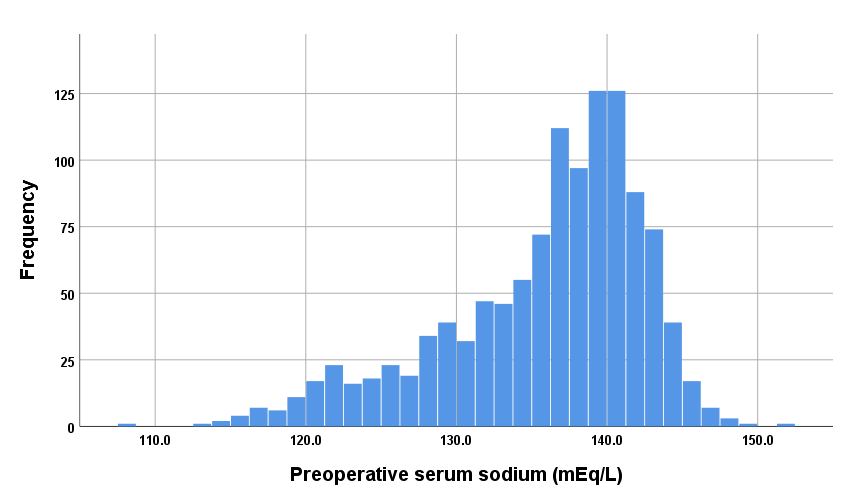


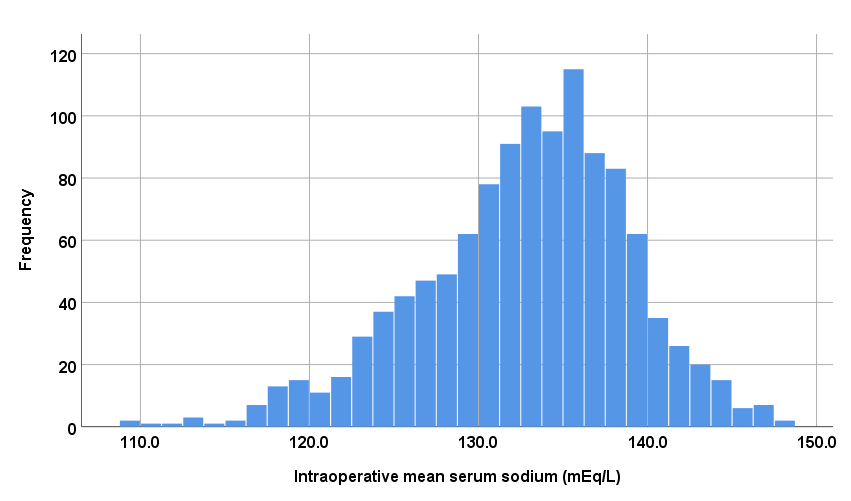


**Supplemental Figure S3.** Kaplan-Meier survival curve analysis according to first intraoperative serum sodium groups (Na <130 mEq/L and 130 ≤ Na ≤ 145 mEq/L) before (upper) and after propensity score matching (lower).


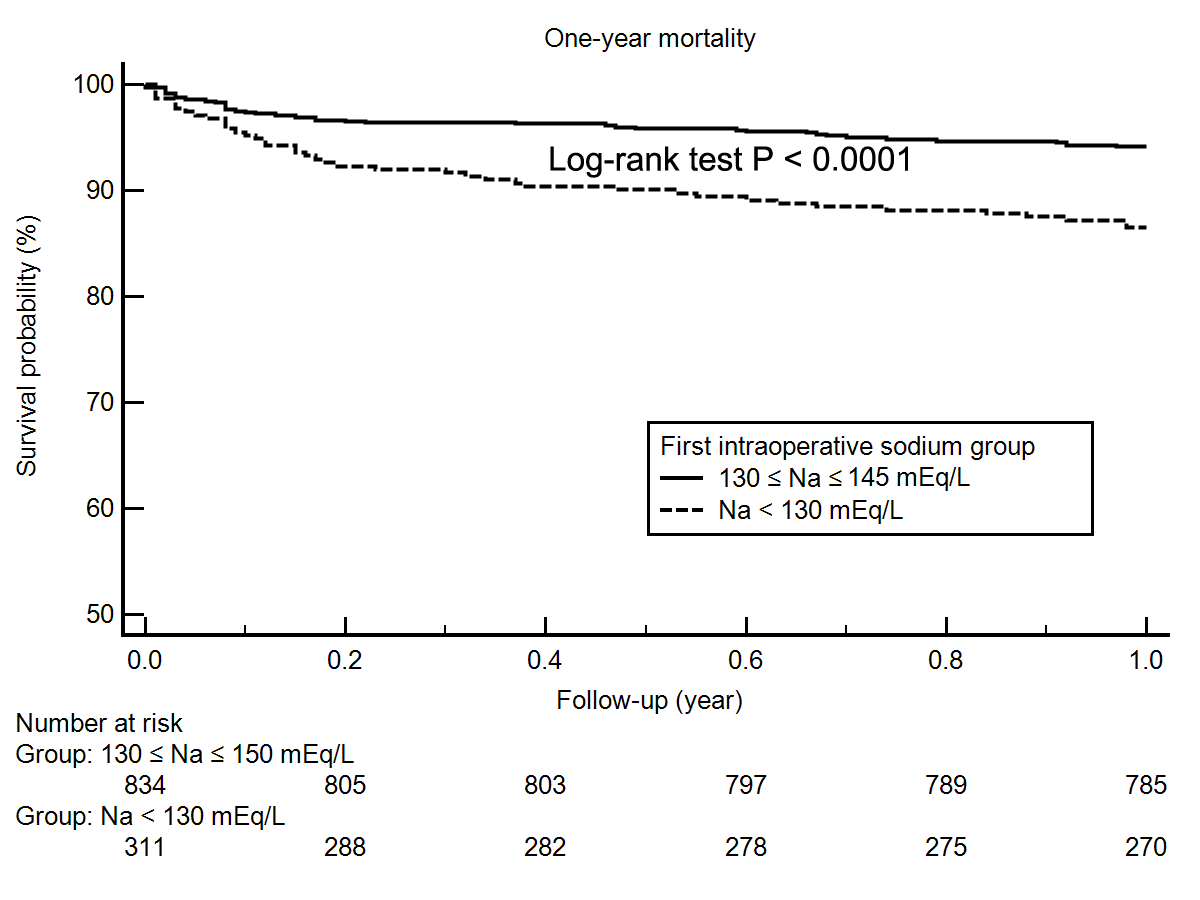


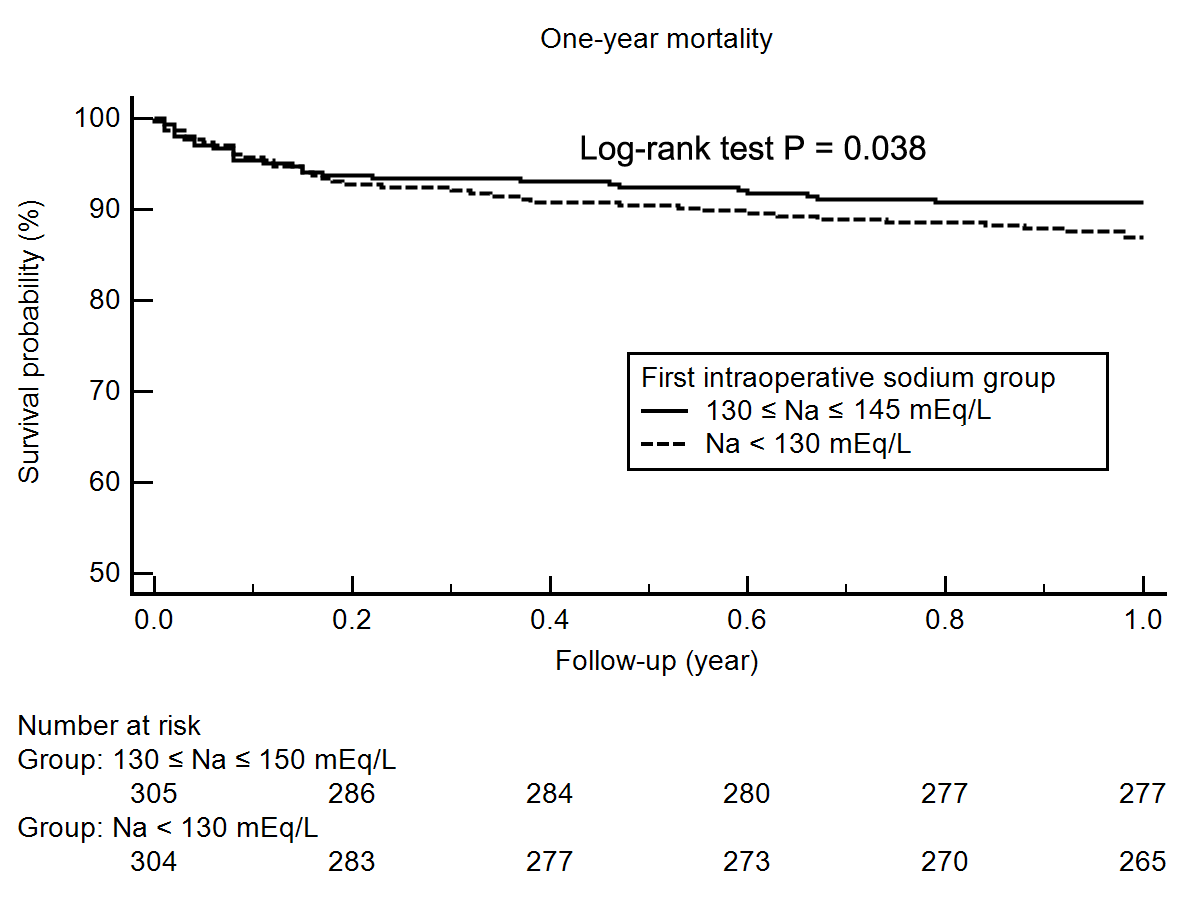


**Supplemental Figure S4.** Line plot and and histograms showing standardized mean differences before and after propensity score matching of two preoperative serum sodium groups (Na <130 mEq/L and 130 ≤ Na ≤ 145 mEq/L). Lower figure shows the covariates balance plot. LDLT_DDLT = living donor liver transplantation versus deceased donor liver transplantation, blood_glucose_averate = intraoperative mean blood glucose, BMI = body-mass index, HTN = hypertension, DM = diabetes mellitus, MELD score = model for end-stage liver disase score, GRWR = graft recipient body-weight ratio, RBC = red blood cell, preop_Hb = preoperative hemoglobin level, preop_Alb = preoperative serum albumin level. Hyperglycemia-corrected sodium levels were use to classify the serum sodium groups.


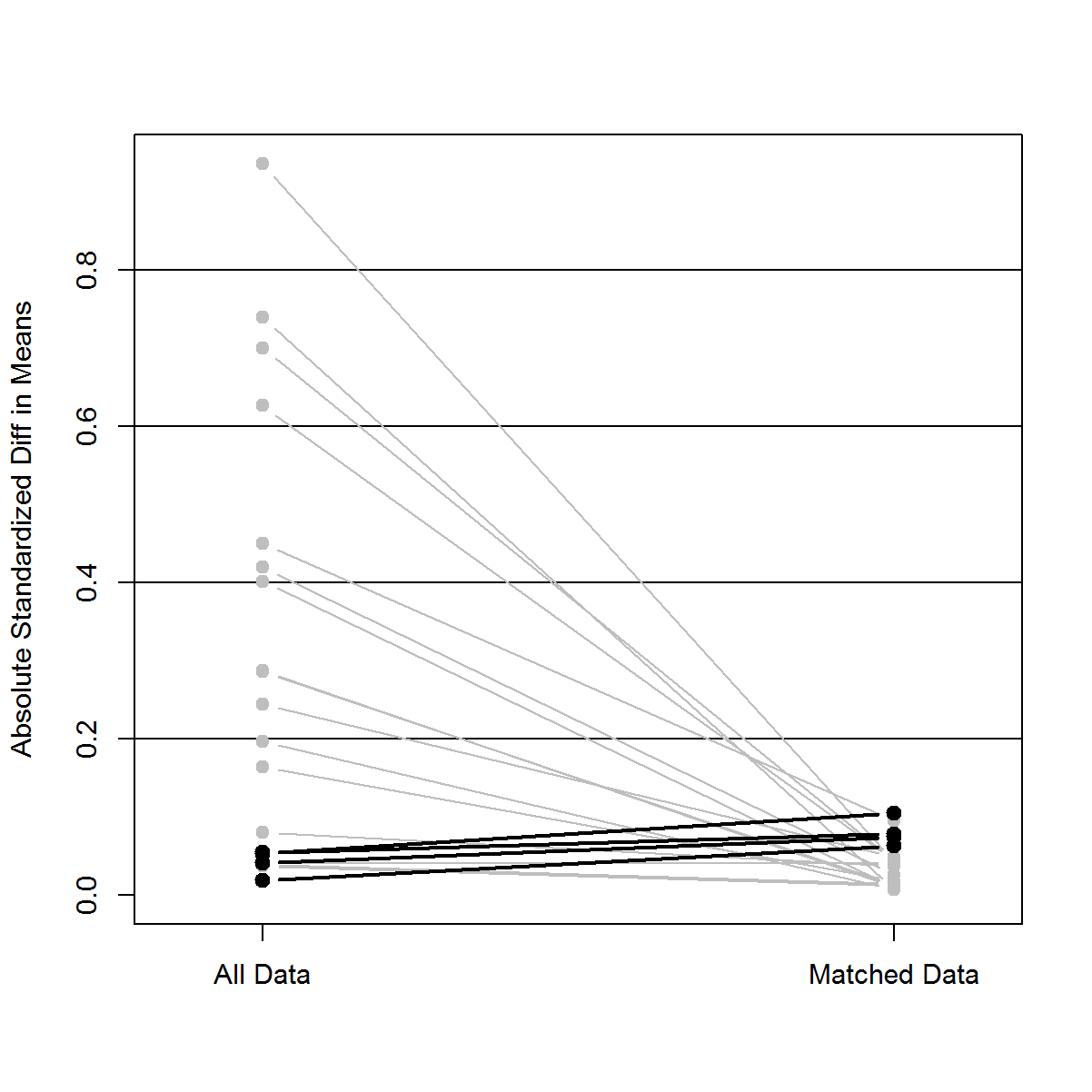


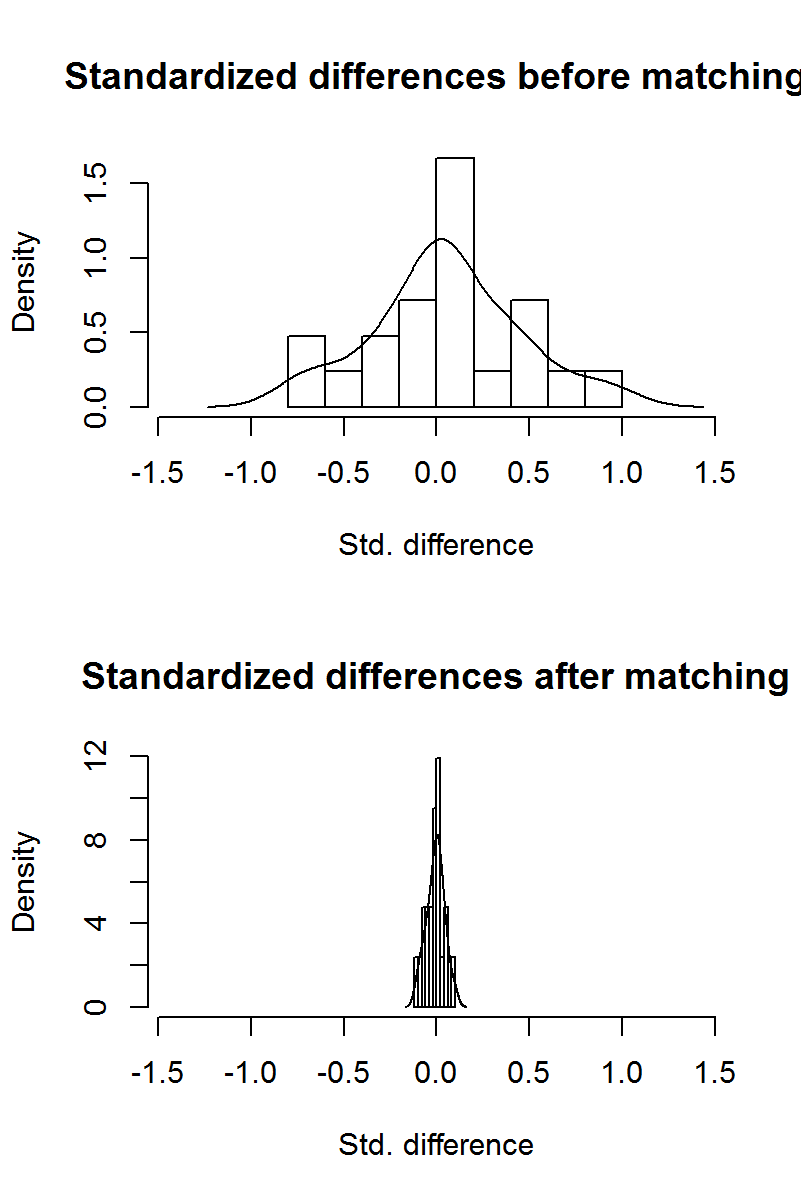

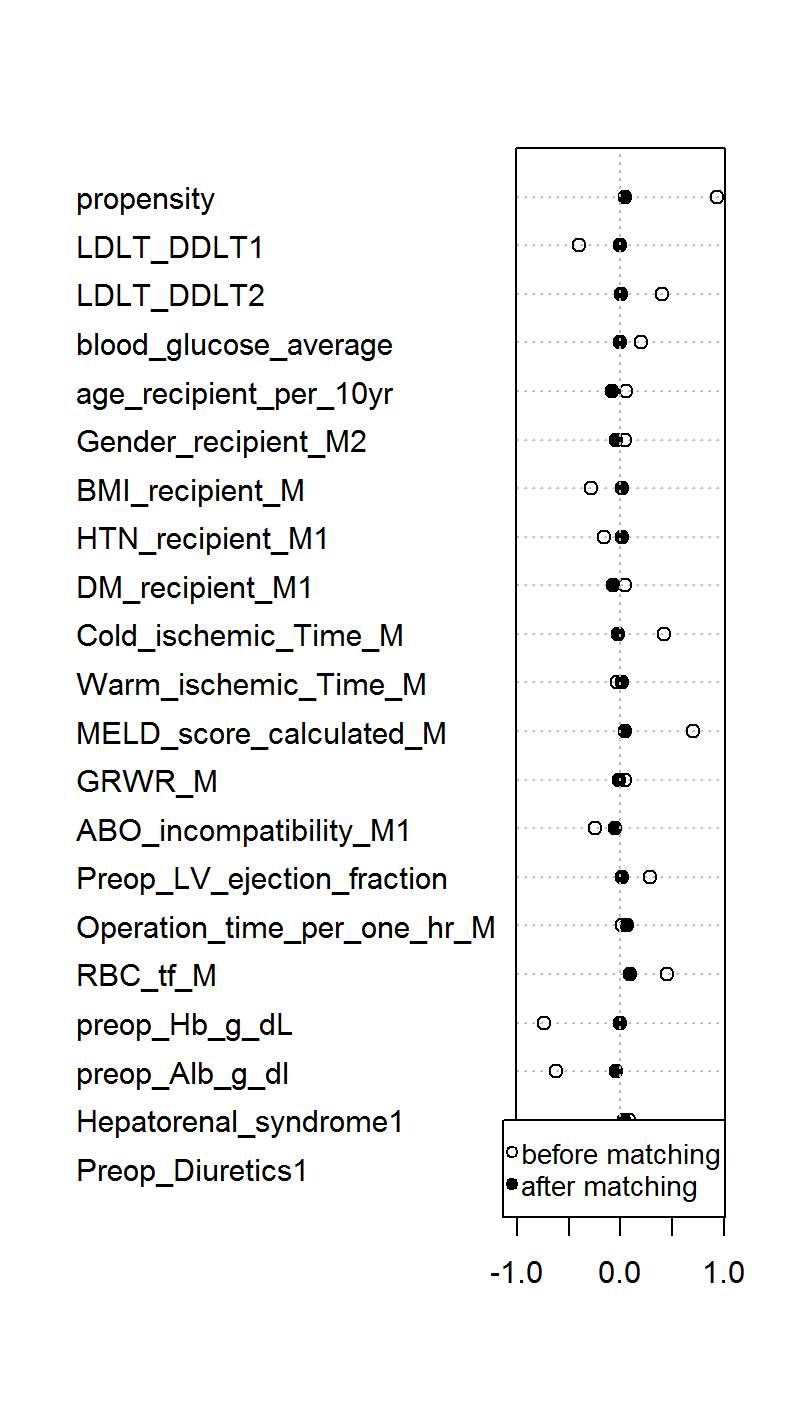


**Supplemental Figure S5.** Line plot and histograms showing standardized mean differences before and after propensity score matching of two intraoperative mean serum sodium groups (Na <130 mEq/L and 130 ≤ Na ≤ 145 mEq/L). Lower figure shows the covariates balance plot. LDLT_DDLT = living donor liver transplantation versus deceased donor liver transplantation, blood_glucose_averate = intraoperative mean blood glucose, BMI = body-mass index, HTN = hypertension, DM = diabetes mellitus, MELD score = model for end-stage liver disase score, GRWR = graft recipient body-weight ratio, RBC = red blood cell, preop_Hb = preoperative hemoglobin level, preop_Alb = preoperative serum albumin level. Hyperglycemia-corrected sodium levels were use to classify the serum sodium groups.


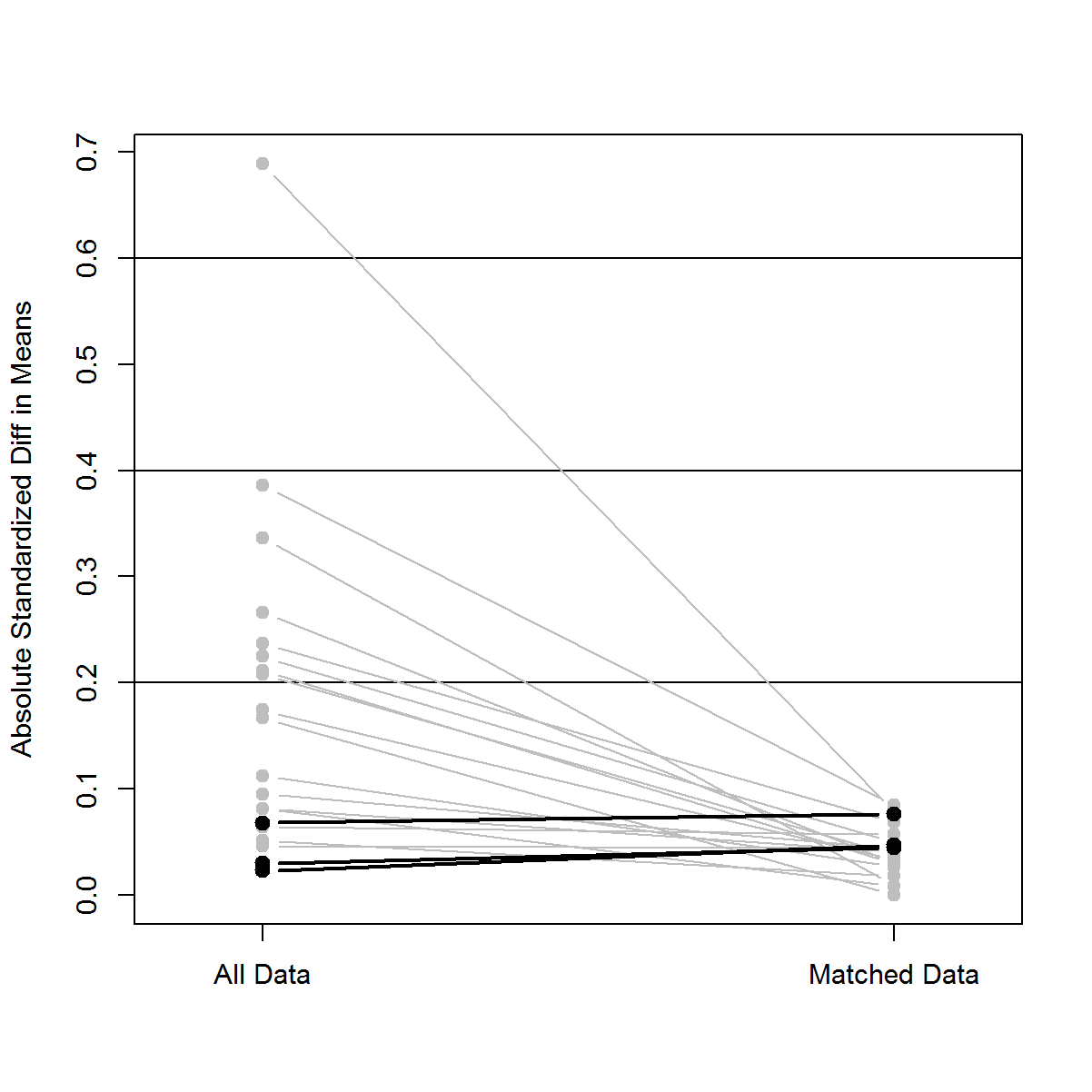


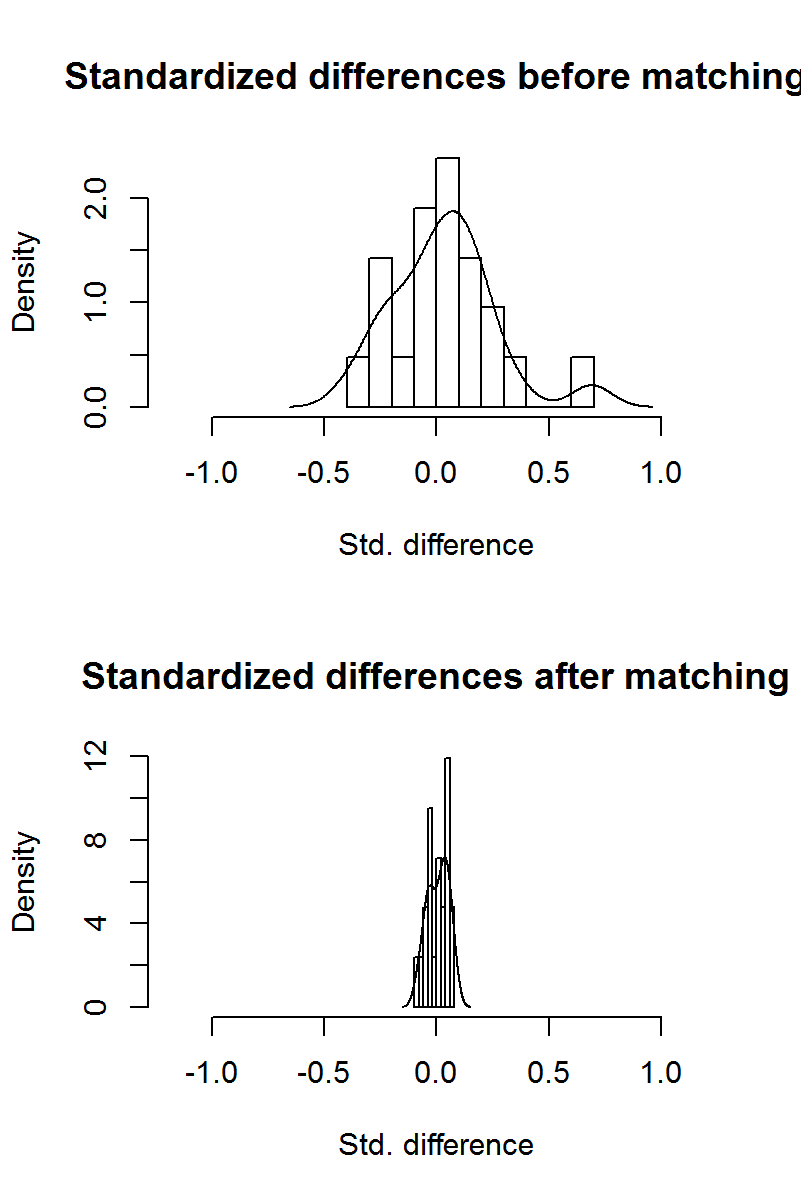

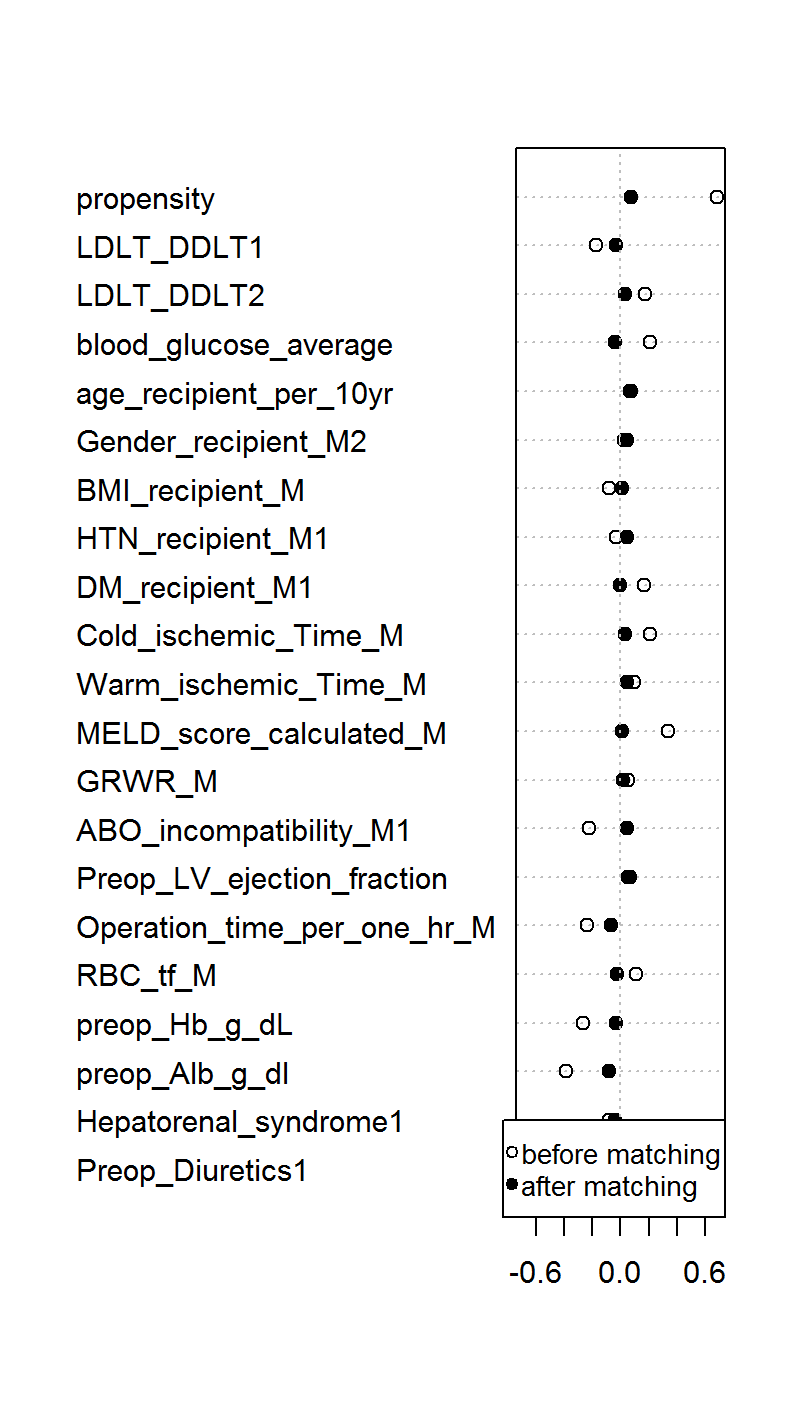

Supplement: Supplementary file 1 — Supplementary materials. [file 41598_2018_37006_MOESM1_ESM.docx]
